# On the Pernicious Effects of Ipecacuanha

**Published:** 1810-03

**Authors:** 


### *To the Editors of the Medical and Physical Journal.*

Gentlemen,

I Lament exceedingly, that your Journal for last June did not earlier attract my notice. If I had been so fortunate, I certainly should have deemed it a duty to have answered with more promptitude Mr. Spencer's statement of the pernicious effects of ipecacuanha upon his apprentice, with the hope that the recital of my own case would

 furnish furnish that gentleman with useful information, and gratify the object of his humane inquiry.

I have preferred this public to any private communication, as best calculated to fulfil a good intention; at the same time, I indulge my inclination to promote the utility of your valuable publication; which so largely contributes to the diffusion of medical knowledge, by recording important or singular facts, illustrative of the history and practice of medicine.

In the year 1787 or 8, when I had been about two years an apprentice, in pounding the root to make the ipecacuanha wine, I was suddenly affected with violent and reiterated sneezings, with a very profuse defluxion from the eyes and nose: these symptoms continued without intermission, for many hours, accompanied by great heat and anguish throughout the cavity of the thorax, and the most oppressive dyspnœa. Exhausted by the violence of this attack, I was conveyed to bed; where supported, for I was unable to lie down, I remained more or less afflicted till the next morning. I arose extremely weakened, and with all the usual appearances of a severe catarrh.

Anterior to this event, I had never been affected by ipecacuanha, or if I had been so, it must have been very slightly; and was perhaps imputed to the sudden effects of an ordinary cold. From this date I have been perpetually tormented by violent catarrhs. Subsequently, the slightest motion of the simple or compound powder of ipecacuanha superinduces precisely similar, but more gentle effects. When weighing or mixing these powders, afterwards, I carefully guarded my mouth and nose by a cloth; but an incautious removal of it, for inspiration, till perhaps half an hour had elapsed, after the medicine was finished, occasioned the same inconveniences. At length I was compelled, except from the most urgent necessity, to quit the shop, while ipecacuanha was in hand; indeed, I have frequently entered my own, or the shop of a stranger, long after it had been used; and by the instant recurrence of these very distressing sensations, have been able too accurately to ascertain the recent exposure of this drug. I have resorted to various expedients for relief, but found none so efficacious as copious draughts of warm water, which appeared to promote expectoration, and with the increase of this, all the symptoms became gradually less irksome.

Although fully aware of the deleterious qualities of ipecacuanha by inspiration, I had no suspicion it would produce, when taken internally, any extraordinary effects.

Some

Some months, perhaps a year, after the first attack, I took a dose of the compound powder of ipecacuanha at bed-time, as a sudorific, in the form of a bolus; I speedily felt the pains in the chest, with dyspnœa, but not so acutely as when introduced by the breath; there was neither sneezing, nor defluxion. Deterred by this uncommon circumstance, I never designedly had recourse to ipecacuanha for more than twenty years. Two accidents lately, within a few weeks of each other, afforded me the opportunity of determining its present effects, when inwardly administered.

A friend, hearing me cough in the street, presented me with a few lozenges; I took two at once; they were scarcely dissolved, ere I felt a pungent roughness in every part of the mouth, exciting a great secretion of saliva; this, it is worthy of noting, was the reverse in the preceding attacks, when the excretory ducts uniformly denied their offices, and occasioned a disagreeable dryness of the mucous membrane. As this acrid sensation extended to the lips, they became prodigiously swollen and inflamed; on the fauces I experienced the like effects, with a most teazing, itching irritation; it descended the trachea, producing pain and dyspnœa; it likewise proceeded down the œsophagus, creating a slight heat in the stomach, and passed with moderate gripings throughout the intestinal canal. The swelling and soreness of the lips continued the longest of these symptoms, but none were sufficiently severe to induce alarm. Soon after, a powder was brought to my house, with an order to prepare more of the same kind. I conveyed a few particles to my tongue, to discover its composition; I quickly experienced those feelings in the mouth and lips, which arose from the lozenges before, but in a milder degree, and they extended no further. Upon referring to the prescription, I found that there was one grain of ipecacuanha, with ten of calcined magnesia. This incident gave birth to an idea, that the former strange affection had originated from the same cause as the latter, and upon due inquiry, my suspicion was confirmed; they were ipecacuanha lozenges which I had swallowed.

This aptitude to take cold, and susceptibility to the action of ipecacuanha varied but little, till I was about thirty years old; since, though still distressed by the application of this drug, yet the violence of its operation is considerably diminished. Catarrhs occur at longer intervals, but with more severity, and with increased difficulty of of breathing, and with more serious derangement of my general health.

In constitutions possessing this peculiar disposition, phthisis pulmonalis will, in all likelihood, supervene, and assuredly still more so, where a spitting of blood may have been excited; happily, this has never happened to me. From a strict adherence to a regular and temporate mode of living, I apprehend no such consequence; but I fear, that at no very distant period, I shall have to contend with a permanent asthma. One of my parents was always extremely liable to catarrh, and at 40 had a severe attack of asthma, from which she has remained entirely free many years; some of my children partake of the same disposition, but neither generation are similarly affected by ipecacuanha.

By the consequences which resulted from the lozenges, it is very probable, that if I had taken as large a dose as is usually exhibited for an emetic, the same train of symptoms would have succeeded, as was witnessed upon Mr. S's apprentice.

Upon a subject so personal and interesting, I have made extensive inquiries, but never heard of more than one other case besides Mr. Spencer's; and that I suspect, is the same as mentioned by Mr. Royston in his Remarks on a Medical Topography. I do not observe that Mr. S's communication and request for information, has received any notice from your numerous readers; hence, it is fair to infer, that this peculiarity of constitution is extremely rare, or surely in so wide a range as your excellent Journal embraces, some practitioner would have transmitted the required intelligence.

The virtues of ipecacuanha are now so justly and universally appreciated, it forms a component part of such various pharmaceutical preparations, prescribed for numerous diseases, that it appears next to impossible, but what the above related singular symptoms must occasionally have occurred in practice. These anomalies would naturally induce considerable surprise and alarm, both in the patient and the medical attendant, who, unsuspicious of the real, would ascribe them to an erroneous cause, which might lead to a very fatal result.

I presume it is highly probable, that these remarkable effects on the system from ipecacuhana, in the first instance, occurred by the excess of a particular stimulus, adventitiously applied to the mucous membrane, which ever after retained a peculiar susceptibility to the specific irritation irritation of this drug; else, why should I have remained nearly two years exposed, yet be insensible to its influence?

Ipecacuanha is capable of being reduced into one of the finest and most impalpable powders; which by motion, is readily diffused, and becomes buoyant in the circumambient atmosphere: these minute particles are soon inhaled with the common air, and produce this simultaneous and irritating action, by contact with the sensitive mucous membrane, extended over the organs of respiration. The grosser particles of snuff, or of other stimulating powders, excite no more irritation on me than on others.

I conceive it will be difficult, if not impracticable, to offer a satisfactory solution to Mr. S's question, "how is this morbid sensibility to be removed?"

For the last eighteen years, I have been little exposed to the effluvia of ipecacuanha; but I know from experience, that as I grow older, its impression, though not lost, is evidently lessened, and, perhaps, will entirely cease with the progress of age.

Gonorrhœa and catarrh are inflammatory affections of the mucous membrane, by the application of certain stimuli. It is undeniable, that from the frequency of the attacks, or from the advance of years, that the aptitude to imbibe, and the violence of both these diseases, materially decline; and at length the habit generally becomes totally insusceptible to the action of either.

Finally, upon these grounds, I conclude, that during the inflammatory diathesis, natural to adolescence, and the earlier stages of life, this peculiar tendency to the morbid effects of ipecacuanha will continue, and that Mr. S's apprentice and myself, can look forward to old age alone for protection; though, if we exist to that period, we shall inevitably be living monuments of this unfortunate and curious idiosyncracy.

I am, &c.

B.

*February* 1, 1810.

*** One of the Editors recollects a somewhat similar effect produced on his father, when exposed to the effluvia of ipecacuanha, whilst it was under bruising. Nothing of the kind, however, happened from the internal use of the same remedy, for Dover's powder was frequently taken with only its common sudorific effect.

*Letter*

